# Aurora-A Kinase as a Promising Therapeutic Target in Cancer

**DOI:** 10.3389/fonc.2015.00295

**Published:** 2016-01-06

**Authors:** Antonino B. D’Assoro, Tufia Haddad, Evanthia Galanis

**Affiliations:** ^1^Department of Medical Oncology, Mayo Clinic College of Medicine, Rochester, MN, USA; ^2^Department of Biochemistry and Molecular Biology, Mayo Clinic College of Medicine, Rochester, MN, USA; ^3^Department of Molecular Medicine, Mayo Clinic College of Medicine, Rochester, MN, USA

**Keywords:** mitotic kinase, cell cycle, cancer, tumor progression, targeted therapy

## Abstract

Mammalian Aurora family of serine/threonine kinases are master regulators of mitotic progression and are frequently overexpressed in human cancers. Among the three members of the Aurora kinase family (Aurora-A, -B, and -C), Aurora-A and Aurora-B are expressed at detectable levels in somatic cells undergoing mitotic cell division. Aberrant Aurora-A kinase activity has been implicated in oncogenic transformation through the development of chromosomal instability and tumor cell heterogeneity. Recent studies also reveal a novel non-mitotic role of Aurora-A activity in promoting tumor progression through activation of epithelial–mesenchymal transition reprograming resulting in the genesis of tumor-initiating cells. Therefore, Aurora-A kinase represents an attractive target for cancer therapeutics, and the development of small molecule inhibitors of Aurora-A oncogenic activity may improve the clinical outcomes of cancer patients. In the present review, we will discuss mitotic and non-mitotic functions of Aurora-A activity in oncogenic transformation and tumor progression. We will also review the current clinical studies, evaluating small molecule inhibitors of Aurora-A activity and their efficacy in the management of cancer patients.

## Introduction

**Figure 1:**
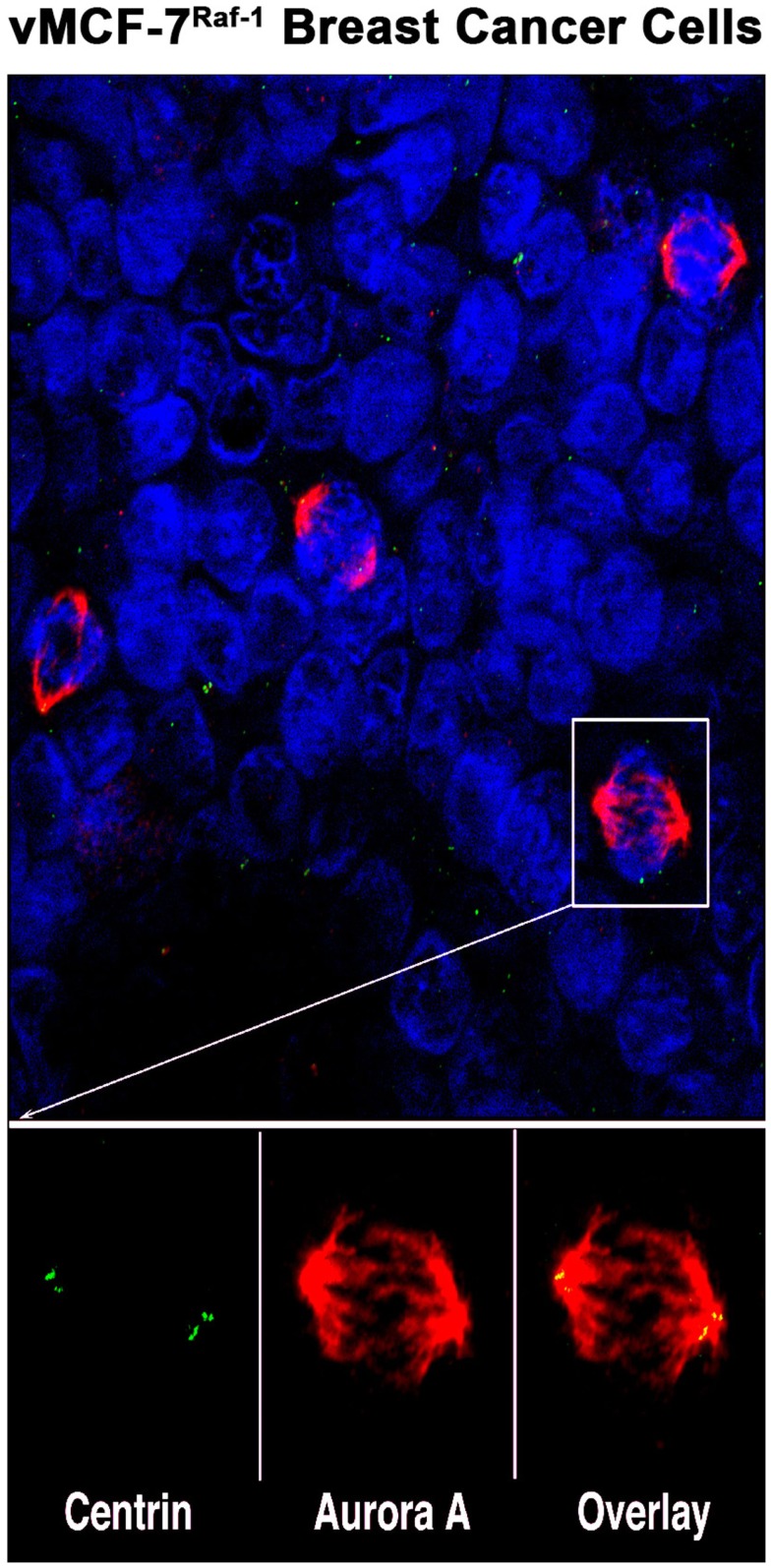
**Aurora-A localization in human breast cancer cells: representative image of mitotic figures from MCF-7 breast cancer cell line engineered to express the Raf-1 oncoprotein (vMCF-7^Raf-1^)**. Centrosomes are labeled in green with 20H5 centrin mouse monoclonal antibody (Mayo Clinic), mitotic spindles are labeled in red with Aurora-A rabbit polyclonal antibody (Abcam, Cambridge, MA, USA) and nuclei are labeled in blue with DAPI (Thermo Fischer Scientific, Rockford, IL, USA). Centrosomal co-localization of Aurora-A is observed in the overlay image (yellow).

Cell division in normal cells is a tightly regulated process by which replicated DNA is equally distributed into two daughter cells (1). Key players that orchestrate cell division are the centrosomes and mitotic spindles that ensure correct chromosome alignment on the metaphase plate and equal chromosome segregation, resulting in the maintenance of a genomic stable diploid karyotype (2). Due to the complexity of the mitotic machinery, several checkpoint surveillance mechanisms have evolved to safeguard accurate temporal and spatial coordination of cell cycle events (3). Abrogation of cell cycle checkpoints impairs the fidelity of correct chromosome segregation and induces chromosomal instability (CIN), a driving force of oncogenic transformation and tumor progression (4, 5). Aurora serine/threonine kinases are key mitotic regulators required for the maintenance of chromosomal stability (6). In mammalian cells, Aurora kinases consist of three members termed Aurora-A, -B, and -C that are expressed in a cell cycle-dependent fashion. These mitotic kinases are highly conserved through evolution and guarantee the precise coordination of cytoskeletal and chromosomal events through modulation of centrosome duplication, maturation, and separation, as well as proper mitotic spindle assembly resulting in equal chromosome distribution into daughter cells (7). While all three Aurora kinases are expressed in human cancer cells, Aurora-A and Aurora-B are best characterized because they are expressed at high levels in aneuploid tumors (8, 9). Aurora-A and Aurora-B share about 70% homology in the carboxyl terminus catalytic domain and three conserved Aurora box motifs in their varying amino terminal domain (10). However, they control cell cycle progression and mitosis by interacting with different proteins. Aurora-A is localized primarily on centrosomes, spindle poles, and transiently along the spindle microtubules as cells progress through mitosis (Figure 1) (11, 12). By contrast, Aurora-B interacts with the chromosomal passenger complex (CPC) that localizes to the inner centromere during prophase through metaphase and then moves to the spindle midzone and the midbody during late mitosis and cytokinesis (13). While some studies have shown that Aurora-B kinase is overexpressed in cancer cells (14, 15), it is not clear whether Aurora-B overexpression is merely associated with the high proliferative activity of cancer cells or if it plays a causative role in tumorigenesis. Due to the lack of definitive evidence that Aurora-B strictly functions as an oncogene, Aurora-A kinase represents a better candidate target for cancer therapeutics. In the last decade, several small molecule inhibitors of Aurora kinases have been developed, though only a few are selective for Aurora-A; they represent promising drugs to impair the progression of aggressive tumors (16).

## Aurora-A Expression in Cancer Cells

The mammalian Aurora-A protein contains 403 amino acids and has a molecular weight of 46 kDa. Aurora-A was first isolated as the product of gene *BTAK* (breast tumor amplified kinase, also named *STK15*) on chromosome 20q13, a region that is frequently amplified in breast, colorectal, and bladder tumors as well as ovarian, prostate, neuroblastoma, and cervical cancer cell lines (17–21). Although gene amplification represents a well-established mechanism to induce Aurora-A overexpression in cancer cells, transcriptional and post-translational mechanisms also play an important role to enhance Aurora-A expression in the absence of *BTAK* gene amplification. In normal cells, the abundance of Aurora-A is down-regulated through APC/C–Cdh1-dependent, proteasome-mediated proteolysis, leading to the organization of the anaphase spindle at the end of mitosis. APC/C–Cdh1-dependent degradation of human Aurora-A requires a destruction box (D-box) in the C-terminal region and a motif in the N-terminus (A-box) (22). Importantly, the phosphorylation state of a serine residue (Ser51) in the A-box inhibits degradation of Aurora-A, as mutants mimicking constitutive phosphorylation of this site cannot be degraded by the APC/C–Cdh1 (23). Furthermore, *we* have showed that HER-2 oncogenic signaling induces Aurora-A phosphorylation, thereby increasing Aurora-A stability and expression in breast cancer cells (24). These findings indicate a functional link between deregulation of Aurora-A stability and tumorigenesis. Conversely, tumor suppressors involved in the control of cell cycle progression promote Aurora-A degradation. The mitotic checkpoint protein Chfr physically interacts with Aurora-A and ubiquitinates Aurora-A both *in vitro* and *in vivo*, ensuring the proper control of mitotic events and maintenance of chromosomal stability (25). Loss of Chfr expression in cancer cells induces aberrant Aurora-A kinase activity, CIN, and promotes tumorigenesis (26). The tumor suppressor p53 modulates Aurora-A expression via both transcriptional and post-translational regulation. Specifically, p53 knockdown in cancer cells promotes the activation of E2F3 transcriptional factor that in turn induces Aurora-A gene expression. p53 deficiency also increases Aurora-A expression through the downregulation of Fbw7α, a key component of e3 ligase of Aurora-A involved in its degradation (27). A separate study demonstrated that highly invasive primary tumors harboring mutant p53 also exhibited Aurora-A overexpression (28). Taken together, these findings strongly demonstrate that Chfr and p53 are key negative regulators of Aurora-A kinase signaling, and their loss of function promotes a growth advantage for cancer cells through increased expression of Aurora-A.

## Aurora-A Promotes Centrosome Amplification, Aneuploidy, and CIN

Aurora-A is overexpressed in a variety of solid tumors, indicative of the critical role that aberrant Aurora-A kinase activity plays in tumorigenesis. Several studies demonstrate the causative function of Aurora-A overexpression in promoting cell transformation *in vitro* and tumor growth *in vivo* employing NIH 3T3 cells and Rat1 fibroblasts (17, 29). The majority of research aims to identify the mechanisms responsible for Aurora-A-induced tumorigenesis has focused on the role of Aurora-A kinase in the control of centrosome duplication and mitosis. Accurate centrosome duplication plays a central role in the maintenance of a normal diploid karyotype. In order to give rise to a bipolar mitotic spindle responsible for the equal segregation of chromosomes to dividing cells, the centrosome must be duplicated once, and only once during each cell cycle (30). Cell cycle checkpoints are essential surveillance mechanisms that guarantee the coordination between centrosome duplication, DNA replication, and mitosis during cell cycle progression (31). Abrogation of cell cycle checkpoints in cancer cells induces centrosome amplification, a pathological condition characterized by the presence of more than two centrosomes within a cell. Centrosome amplification may result from inactivation of the G1/S checkpoint leading to centrosome overduplication or from abrogation of the G2/M checkpoint leading to cytokinesis failure, endoreduplication, and consequent centrosome accumulation (2). Centrosome amplification due to cytokinesis failure is exacerbated in cancer cells lacking the “G_1_ phase post-mitotic checkpoint” that is dependent on the integrity of p53/Rb axis (32–34). One of the major consequences of centrosome amplification is the formation of multipolar or pseudo-bipolar mitotic spindles that will result in unequal chromosome segregation and aneuploidy (35–37). Aneuploidy is characterized by gains and/or losses of whole chromosomes during cell division and occurs in early stages of tumor development, playing a critical role in both tumorigenesis and tumor progression (38). Significantly, while aneuploidy represents the state of an aberrant karyotype, the continuous generation of chromosome variations in cancer cells is defined as CIN that will ultimately drive genetic heterogeneity, tumor recurrence, and poor outcome (39). Several lines of evidence have established that centrosome amplification drives CIN and genetic heterogeneity in aneuploid tumors (40–42). Elegant studies have demonstrated that deregulated expression of Aurora-A is functionally linked to centrosome amplification and CIN (43–45). The major mechanism by which aberrant Aurora-A kinase activity induces centrosome amplification and CIN is through cytokinesis failure and consequent multinucleation leading to centrosome accumulation (46). Aurora-A induces cytokinesis failure and centrosome amplification mainly through its interaction with key tumor suppressor gene products that control cell cycle checkpoints, centrosome duplication, and chromosomal stability. Aurora-A phosphorylates the tumor suppressor p53 on *Ser^*215*^* residue, abrogating the DNA-binding and transactivation activity of p53 that results in the inhibition of the downstream target gene p21 involved in the control of centrosome duplication (47). Moreover, Aurora-A-mediated phosphorylation of p53 on *Ser^*315*^* residue will increase the affinity of p53 with Mdm2 that in turn will promote p53 degradation (48). The tumor suppressors BRCA1 and BRCA2 play a central role in the maintenance of chromosomal stability and germline mutations in BRCA1 and BRCA2 genes have been detected in approximately 90% of hereditary breast/ovarian cancers (49). Specifically, BRCA1 monitors the physical integrity of DNA following genotoxic stress and coordinates DNA replication with centrosome duplication cycle (50). It has been demonstrated that Aurora-A directly binds to BRCA1 and phosphorylates it on *Ser^*308*^* residue. Deregulated Aurora-A-mediated BRCA1 phosphorylation on *Ser^*308*^* residue induces abrogation of the G2/M checkpoint leading to centrosome amplification and CIN (51). Moreover, Aurora-A is required to activate polo-like kinase 1 (PLK1) that plays a key role in promoting centrosome duplication and mitotic entry (52, 53). These findings indicate that Aurora-A overexpression induces aberrant Plk1 activity that will drive centrosome amplification, improper segregation of chromosomes, CIN, and tumorigenesis. Leontovich et al. uncovered a novel mechanism by which Cyclin-A/Cdk2 oncogenic signaling favors Aurora-A centrosomal localization that in turn induces centrosome overduplication in breast cancer cells (54). Taken together, these studies strongly demonstrate that deregulated Aurora-A kinase activity induces centrosome amplification in cancer cells through different mechanisms and results in the development of CIN, a driving force for genetic heterogeneity and tumor progression.

## Non-Mitotic Function of Aurora-A in Tumorigenesis

Although Aurora-A-mediated centrosome amplification and CIN represents a well-recognized mechanism that promotes oncogenic transformation, the kinase activity of Aurora-A is essential to acquire a transformed phenotype regardless of the induction of centrosome amplification (55). These findings led to the discovery that Aurora-A kinase also phosphorylates proteins unrelated to centrosome function that play a central role in tumorigenesis. Taga et al. showed in U2OS human osteosarcoma cells that Aurora-A induces phosphorylation of Akt and mTOR oncoproteins that is required to increase U2OS tumorigenicity (56). In agreement with these results, aberrant Aurora-A kinase activity promotes resistance to cisplatin, etoposide, and paclitaxel-induced apoptosis by phosphorylating Akt in wild-type p53 ovarian cancer cells (57). Other studies have revealed the direct role of Aurora-A kinase activity in mediating cancer cell motility and distant metastases. Aurora-A promotes breast cancer metastases by dephosphorylation of cofilin and activation of cofilin–F-actin pathway, which accelerates actin reorganization and polymerization (58). Furthermore, inhibition of phosphatidylinositol 3-kinase (PI3K) oncogenic signaling blocked Aurora-A-mediated cofilin dephosphorylation, actin reorganization, and cell migration. These results uncover a novel crosstalk between PI3K signaling and Aurora-A in tumor progression. In esophageal squamous cell carcinoma cells, Aurora-A overexpression induces cell migration and invasion as well as secretion and expression of matrix metalloproteinase-2 (MMP-2). This mechanism is mediated by Aurora-A-induced phosphorylation of p38 MAPK and Akt protein kinases (59). Aberrant Aurora-A kinase activity also induces activation of Rap1, a member of the Ras family of small GTPases, leading to the development of distant metastases originating from oral cavity squamous cell carcinomas (60). Du and Hannon demonstrated that Aurora-A kinase activity inhibits the function of Nm23-H1 protein that is involved in the suppression of distant metastases, facilitating tumor progression (61).

Moreover, recent studies revealed a novel function of Aurora-A in the progression of solid tumors through activation of epithelial–mesenchymal transition (EMT) and stemness reprograming. Cammareri et al. demonstrated that Aurora-A overexpression is restricted in colorectal cancer stem cells (CR-CSC), and Aurora-A inhibition restored chemosensitivity and compromised the tumor initiating ability of CR-CSC to form tumor xenografts in immunocompromised mice (62). The causative role of Aurora-A overexpression in promoting EMT and tumor progression through stabilization of Snail transcription factor has been shown in head and neck cancer cells (63). Significantly, we have defined for the first time the essential role of Aurora-A in promoting breast cancer progression through activation of EMT and the genesis of breast cancer stem cells responsible for the onset of distant metastases (24). Moreover, Aurora-A-induced EMT and onset of distant metastases was functionally linked to SMAD5 and SOX2 expression, two master transcription factors involved in the development of EMT, tumor self-renewal, and an invasive, basal-like phenotype. In the same study, we have uncovered the causative role of Aurora-A overexpression in inducing expansion of cancer stem cells through impairment of asymmetric divisions. These results are in agreement with a previous study showing that a phosphorylation cascade triggered by the activation of Aurora-A kinase is responsible for the asymmetric localization of Numb during mitosis (64). Taken together, these studies highlight an essential role of Aurora-A kinase in driving tumor progression by modulating the activity of key oncogenic pathways involved in cell migration, chemoresistance, tumor initiating ability, and onset of distant metastases.

## Aurora-A as a Novel Biomarker Prognostic of Poor Clinical Outcome

Several studies have shown that Aurora-A kinase is overexpressed in a variety of tumors, suggesting that Aurora-A may represent a promising prognostic biomarker. Reiter et al. reported that increased expression of Aurora-A in head and neck squamous cell carcinomas was significantly associated with shorter disease-free and overall survival of patients (65). Likewise, Aurora-A overexpression is associated with centrosome amplification and shorter survival in an extensive proportion of ovarian tumors (66, 67). Gastrointestinal tumors also display deregulation of Aurora-A expression that is linked to high risk of recurrence and tumor progression. Employing tissue microarrays from a retrospective cohort of 343 patients with colorectal cancer liver metastases, Goos et al. showed that Aurora-A levels were increased in liver metastatic lesions compared to corresponding primary tumors and was associated with poor clinical outcome (68). Wang et al. showed that Aurora-A overexpression was an independent prognostic marker of poor survival in gastric cancer patients without lymph node metastases (69). Samaras et al. performed a comparative immunohistochemical analysis of Aurora-A and Aurora-B expression in 40 patients with primary glioblastomas to identify possible correlations with Ki-67 proliferation index and clinical outcomes (70). While Aurora-A was overexpressed in glioblastomas with high Ki-67 expression and was associated with poor survival, Aurora-B expression was not correlated with Ki-67 expression and patient survival. Aurora-A overexpression has also been established as a valuable biomarker prognostic of poor clinical outcome in breast carcinomas. Nadler et al. demonstrated in a tissue microarray containing primary breast tumor tissue from 638 patients with 15-year follow-up that aberrant expression of Aurora-A, but not Aurora-B, was an independent prognostic marker strongly correlated with decreased survival (71). High Aurora-A expression was also associated with high nuclear grade and elevated HER-2/neu and progesterone receptor expression. In 48 cases of operable triple-negative breast tumors, Yamamoto et al. established that basal-like subtype was significantly associated with high levels of Aurora-A and shorter disease-free and overall survival compare to non-basal-like breast tumors (72). Using microarray-based gene expression data from three independent cohorts of 766 node-negative breast cancer patients, Siggelkow et al. demonstrated that patients harboring high Aurora-A expression had a shorter metastasis-free survival in the molecular subtype estrogen receptor-positive (ER+)/HER2− carcinomas, but not in ER−/HER2− or HER2+ carcinomas (73). A recent study reported, in a cohort of 426 patients with primary breast cancer, that elevated expression of Aurora-A and SURVIVIN, together with *BTAK* gene amplification, is correlated with increased CIN and shorter survival (74). Taken together, these studies highlight Aurora-A as a novel, independent prognostic biomarker of poor clinical outcome that could identify patients at high risk of tumor recurrence or progression.

## Pharmacologic Targeting of Aurora-A Kinase Activity in Cancer Therapy

In the last decade, at least 13 different inhibitors of the Aurora kinases have been evaluated in phase I clinical trials in patients with various hematologic and solid tumor malignancies. Nearly all of the initial agents studied were pan-inhibitors of Aurora-A, -B, and -C, and several of them furthermore inhibited other kinases, such as bcr–abl (T135I), Flt3, VEGFR2, and JAK 2/3. Some of these trials were suspended and not completed or published. Some inhibitors have not continued beyond phase I evaluation due to significant toxicities at clinically effective doses or limited clinical antitumor activity. Only a limited number of these pan-Aurora and multi-kinase inhibitors have been pursued in phase II clinical trials (AT-9283, MK-0457, ENMD-2076, PHA-739358). Three of the Aurora kinase inhibitors developed were selective for Aurora-A (MLN 8054, MLN 8237, TAS-119). Of all the inhibitors, only MLN 8237 (alisertib) has proceeded to phase III evaluation.

The first of the selective Aurora-A kinase inhibitors to enter into human studies was MLN 8054. In Phase I dose escalation studies in patients with advanced solid cancers, the observed dose limiting toxicity (DLT) was reversible somnolence, attributed to GABA_A_ α-1 benzodiazepine off-target binding (75, 76). With the aim of improving the therapeutic window, the chemical structure of the molecule was modified, and of potential new agents, MLN 8237 (alisertib) was selected for further development based on preclinical evidence demonstrating its increased potency in Aurora-A enzymatic inhibition, reduced degree of brain partitioning, and while GABA_A_ binding potency was comparable to MLN 8054, alisertib displayed a greater selectivity ratio of Aurora-A inhibition to GABA_A_ α-1 benzodiazepine site binding affinity (77).

In 2007, the first clinical trial opened to evaluate alisertib, an orally administered, small molecule inhibitor that is selective for Aurora-A kinase. To date, well over 1000 patients with hematological or solid tumor malignancies have participated in clinical trials with the agent as monotherapy or in combination with chemotherapy or other targeted agents (78, 79). In the original phase I trials, different formulations of the drug, doses, and schedules were evaluated (80, 81). Stomatitis and neutropenia were the most common DLTs consistent with its antiproliferative effect. Somnolence was evident in patients receiving once daily dosing of alisertib at the highest dose levels; however, the frequency and severity of these episodes were reduced with twice daily dosing of alisertib at lower individual doses, which reduced peak plasma levels while maintaining overall systemic exposures. Other common low-grade toxicities included alopecia, nausea, diarrhea, anemia, and fatigue. The recommended phase II dose was 50 mg twice daily on days 1–7 of a 21-day cycle, and the preferred formulation was the enteric-coated tablet; both were confirmed in the industry-sponsored study of alisertib as monotherapy in patients with advanced solid tumor malignancies (82). Encouraging clinical activity was demonstrated in this trial. In the cohort of heavily pre-treated women with hormone receptor-positive metastatic breast cancer (*n* = 26), 23% had an objective response (complete or partial response) and 31% achieved stable disease for at least 6 months, resulting in a clinical benefit rate of 54%. Median PFS was 7.9 months. In the chemotherapy-refractory, relapsed small cell lung cancer (SCLC) cohort (*n* = 12), a response rate of 25% was observed with a median duration of response of 4.3 months. A phase II trial of alisertib alone or combined with paclitaxel for second-line therapy of SCLC is currently active (NCT02038647). Based on promising activity observed in relapsed/refractory peripheral T-cell lymphoma (83, 84), a phase III clinical trial of alisertib versus treatment of investigator’s choice (NCT01482962) was pursued but subsequently terminated enrollment at a pre-specified interim analysis due to projections that the study was unlikely to meet the primary endpoint of superior PFS.

An alternative 28-day regimen with alisertib given days 1–3, 8–10, and 15–17 was studied in combination with paclitaxel in breast and ovarian cancer models, and it is associated with equivalent drug levels, decreased incidence of dose limiting neutropenia with negligible compromise to efficacy (85). The safety and tolerability of this schedule in combination with fulvestrant is currently being explored in an ongoing phase I trial in patients with hormone receptor-positive, advanced breast cancer (NCT 02219789).

TAS-119 is the only other selective Aurora-A kinase inhibitor to enter into clinical evaluation. It is being studied as monotherapy and in combination with taxane-based chemotherapy in two separate, active phase I clinical trials (NCT02134067, NCT02448589).

## Conclusion

**Figure 2:**
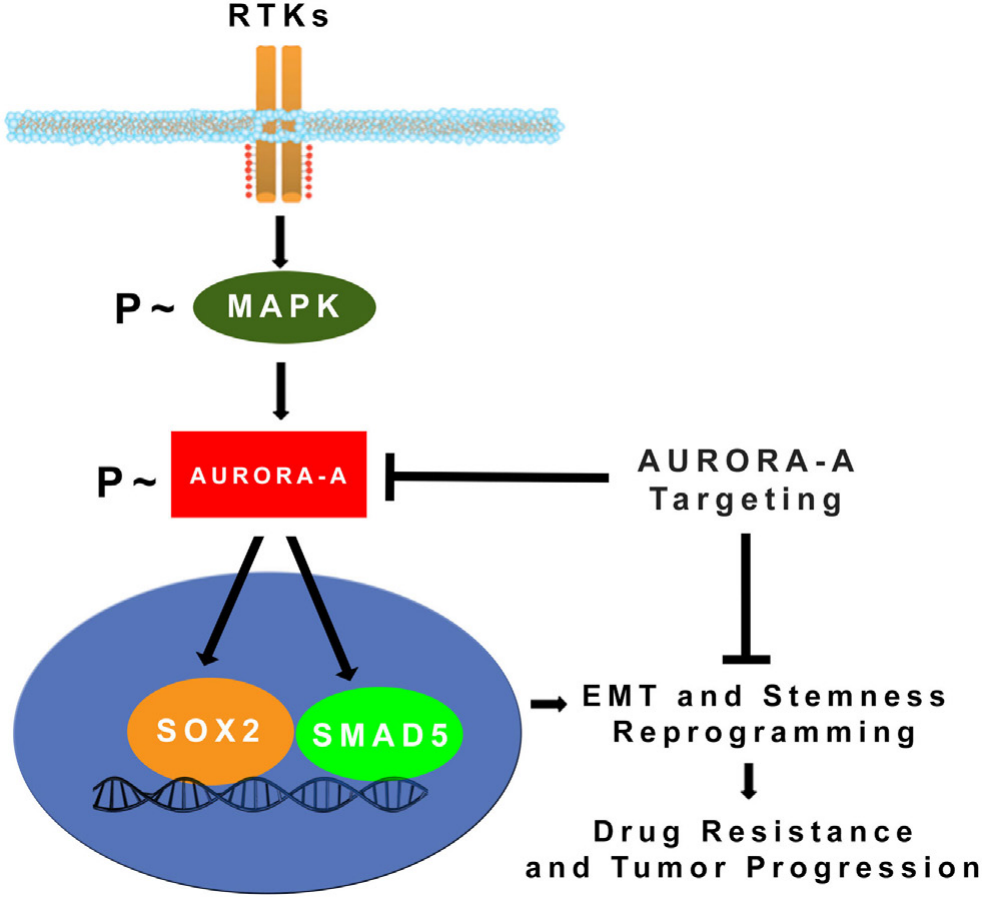
**MAPK-induced activation of Aurora-A kinase promotes EMT, stemness, and tumor progression: constitutive activation of MAPK oncogenic signaling during tumor growth leads to stabilization and accumulation of Aurora-A kinase**. Aberrant Aurora-A kinase activity induces activation of SMAD5 and SOX2 transcription factors that in turn will orchestrate EMT and stemness reprograming leading to drug resistance and tumor progression (24). Pharmacologic targeting of Aurora-A kinase activity can be effective for eliminating highly invasive cancer stem cells and defeat tumor progression.

One of the major hallmarks of cancer is aneuploidy and the development of CIN characterized by the relentless generation of chromosome variations that will ultimately drive genetic heterogeneity and tumor progression. In normal cells, cell division is monitored by checkpoints that are safeguard mechanisms to guarantee the accurate temporal and spatial coordination of cell cycle events. Abrogation of cell cycle checkpoints induces centrosome amplification that impairs the fidelity of correct chromosome segregation, promoting aneuploidy and CIN. Members of the Aurora serine/threonine kinase family are key mitotic regulators required for the maintenance of chromosomal stability. Overexpression and aberrant activation of Aurora-A kinase has been functionally linked to oncogenic transformation mainly through development of centrosome amplification and CIN. Significantly, recent studies have also demonstrated that Aurora-A kinase mediates MAPK-induced distant metastases through activation of EMT and stemness reprograming (Figure 2). Taken together, these findings demonstrate that Aurora-A kinase represents a critical “*druggable target*” in cancer, controlling key oncogenic pathways associated with drug resistance and poor patient outcome. For this reason, several small molecule inhibitors of Aurora-A kinase activity have been developed and their efficacy is being tested in clinical trials (Table 1). Significantly, recent studies have highlighted the incremental therapeutic efficacy when combining Aurora-A inhibitors with conventional anti-cancer drugs to restore chemosensitivity and inhibit tumor progression, a strategy expected to further build on the clinical benefit potential of Aurora-A inhibition.

**Table 1:** **Aurora kinase inhibitors in clinical trials**.

	Inhibitor commercial name	Clinical trials
Pan-Aurora inhibitors	VX-680/MK-0457 (Vertex/Merck) Tozasertib	Phase II (terminated due to toxicity)
	PHA-739358 (Pfizer/Nerviano) Danusertib	Phase II
	PHA-680632 (Pfizer/Nerviano)	Phase I
	CYC-116 (Cyclacel)	Phase I
	SNS-314 (Sunesis)	Phase I
	R763 (Rigel)	Phase I
	AMG-900 (Amgen)	Phase I
	AT-9283 (Astex)	Phase II
	PF-03814375 (Pfizer)	Phase I
	GSK1070916 (GlaxoSmithKline)	Phase I
Aurora-A inhibitors	MLN8237 (Millennium)	Phase II
	ENMD-2076 (EntreMed)	Phase II
	MK-0457 (Vertex)	Phase II

## Conflict of Interest Statement

The authors declare that the research was conducted in the absence of any commercial or financial relationships that could be construed as a potential conflict of interest.
